# Overexpression of a disintegrin and metalloproteinase 9 (ADAM9) in relation to poor prognosis of patients with oral squamous cell carcinoma

**DOI:** 10.1007/s12672-024-01422-1

**Published:** 2024-10-23

**Authors:** Shuangjiang Wu, Lang Cheng, Tao Luo, Anupong Makeudom, Lei Wang, Suttichai Krisanaprakornkit

**Affiliations:** 1 Department of Oral and Maxillofacial Surgery, The Affiliated Hospital, Southwest Medical University, Luzhou, China; 2 Department of Oral and Maxillofacial Surgery, The Affiliated Stomatological Hospital, Southwest Medical University, Luzhou, China; 3 Luzhou Key Laboratory of Oral & Maxillofacial Reconstruction and Regeneration, The Affiliated Stomatological Hospital of Southwest Medical University, Luzhou, China; 4 School of Stomatology, Southwest Medical University, Luzhou, China; 5 Department of Pathology, The Fifth Hospital of Deyang, Deyang, China; 6 School of Dentistry, Mae Fah Luang University Medical Center, Mae Fah Luang University, 365 Moo 12, Nang Lae Subdistrict, Mueang Chiang Rai District, Chiang Rai, 57100 Thailand

**Keywords:** A disintegrin and metalloproteinase 9, CUB domain-containing protein 1, Oral squamous cell carcinoma, Overexpression, Tissue-type plasminogen activato

## Abstract

This study investigates the expressions of ADAM9, CDCP1 and t-PA in OSCC and their impacts on patient prognosis. Previous research has demonstrated the overexpression of ADAM9 and activation of plasminogen activator in OSCC, but CDCP1's role remains unexplored. While these biomolecules are known to contribute to lung cancer metastasis, their concurrent expressions in OSCC have not been thoroughly examined. Our aim is to assess the expressions of ADAM9, CDCP1, and t-PA in OSCC specimens, compare them with normal oral tissues, and explore their correlation with OSCC's clinicopathological features and patient survival outcomes.

**Supplementary Information:**

The online version contains supplementary material available at 10.1007/s12672-024-01422-1.

## Introduction

OSCC is the most common oral cancer, accounting for more than 80% of oral and maxillofacial malignancies [1]. Currently, the five-year survival rate for patients with OSCC remains low [2]. This highlights a significant gap in our understanding of the disease's underlying biological mechanisms, which is crucial for developing more effective targeted therapies [3]. Improving the prognosis for OSCC patients poses a difficult and ongoing challenge in clinical intervention [4]. Thus, identifying and exploring novel molecular targets related to OSCC is of great clinical significance, as it could pave the way for more effective therapeutic strategies.

ADAMs consist of 33 members that regulate a number of cellular processes, such as, cell fusion, adhesion, and migration [5]. As a member of the ADAM family, ADAM9 is involved in the occurrence, progression, invasion, metastasis, and prognosis of liver cancer, breast cancer, lung cancer, stomach cancer, kidney cancer, prostate cancer, and other malignant tumors [6–8]. Zhang et al. studied the abnormal expression of ADAM9 in human resected non-small cell lung cancer and found that, compared with normal lung tissues, ADAM9 was highly expressed in lung tumors, and ADAM9 overexpression was an independent factor for shortened survival time [9, 10]. Wang et al. reported that the abnormal expression of ADAM9 in gastric cancer tissues was associated with different clinicopathological features [11], which is consistent with previous studies on other solid tumors [12, 13]. Liu et al. found that miR-129-5p played a tumor suppressor role in the progression of gastric cancer by targeting ADAM9 [14]. ADAM9 overexpression in solid tumors is also associated with aggressive tumor phenotype and poor clinical prognosis [15–17]. In addition, ADAM9 silencing can delay the establishment of Dabrafenib resistance [18]. Our research group has previously reported ADAM9 overexpression in oral cancer [19], and this study further explored its clinicopathological significance.

CDCP1 is a transmembrane glycoprotein that is not cleaved by serine proteases under normal physiological conditions but can be cleaved in response to tumor or tissue damage [20]. Dysregulation of CDCP1 expression has been associated with multiple cancers, including leukemia, metastatic colon cancer, and breast cancer [21]. Awakura et al. [22] and Ikeda et al. [23] found that CDCP1 was highly expressed in renal cancer and lung adenocarcinoma, the expression of CDCP1 was significantly correlated with the development of the disease, and patients with high expression of CDCP1 had a lower overall survival rate. Lin et al. [24] found that ADAM9 activity affected the expression of CDCP1. Chiu et al. [25] found that ADAM9 enhanced the expression of CDCP1 and promoted lung tumor metastasis by inhibiting miR-218. In a subsequent study, they proposed that ADAM9 inhibits miR-1 through activation of epidermal growth factor receptor [26], thereby enhancing the role of CDCP1 in lung cancer metastasis.

t-PA is a serine protease responsible for degrading blood clots by converting plasminogen into plasmin. Lin et al. [24] observed that high t-PA expression in human lung adenocarcinoma cells was associated with tumor metastasis to the brain. Moreover, decreased ADAM9 levels in ADAM9 knockout cells significantly reduced CDCP1 and t-PA expressions. In addition, cleavage of CDCP1 was weakened in t-PA knockdown cells, while cleavage of CDCP1 was slightly increased after high-dose t-PA treatment. Further analysis showed that t-PA activity was highly correlated with CDCP1 cleavage in these cancer cells; thus, t-PA is responsible for CDCP1 activation. All of these results suggest that ADAM9 enhances CDCP1 cleavage mediated by t-PA activation. However, the ADAM9/CDCP1/t-PA pathway and their aberrant expressions have so far been human lung adenocarcinoma cell lines and lung cancer. Whether these molecules are clinically significant in OSCC needs to be further investigated.

The aim of this study was to examine the degrees of ADAM9, CDCP1, and t-PA expressions in OSCC by immunohistochemistry and to explore the correlations of ADAM9, CDCP1, and t-PA expression levels with the clinicopathological features of OSCC. Our findings revealed that the expression levels of ADAM9, CDCP1, and t-PA in OSCC specimens were significantly different from those in normal oral tissues. Furthermore, we found meaningful correlations between the expressions of these biomarkers and the clinicopathological features of OSCC, as well as with patient survival outcomes. These results suggest that ADAM9, CDCP1, and t-PA could serve as potential biomarkers for early diagnosis and as targets for novel therapeutic strategies, ultimately improving the prognosis of OSCC patients.

## Materials and methods

### Acquisition of normal oral mucosal and OSCC specimens

A total of 63 patients diagnosed with OSCC, excluding lip cancer, which has a higher survival rate than other types of oral cancer [27], were recruited from the Department of Oral and Maxillofacial Surgery, the Affiliated (Stomatological) Hospital of Southwest Medical University, Luzhou, China, under an approval of its Medical Ethics Committee (#20190215001). Written informed consent was obtained from all patients. All patients underwent extended resection of oral cancer with the surgical margin away from the tumor at least 2 cm, neck lymph node dissection, and flap reconstruction, between 2019 and 2022. Relevant clinical and pathological data, including gender, age, pTNM staging, degree of tumor differentiation, and pathological diagnosis, were obtained. Follow-up data included patients' health status (disease progression, treatment, recurrence, and time of death, etc.). The clinical characteristics of 63 OSCC cases are summarized in Table 1.

**Table 1:** Clinical features of 63 OSCC cases and twelve healthy donors

	Normal tissues	Well differentiated	Moderately differentiated	Poorly differentiated
Gender	12	28	24	11
Male	7 (58.3%)	22 (78.6%)	18 (75.0%)	8 (72.7%)
Female	5 (41.7%)	6 (21.4%)	6 (25.0%)	3 (27.3%)
Age				
Min	31	28	20	32
Max	80	77	80	73
Mean ± SD	60.250 ± 13.639	59.964 ± 14.232	59.750 ± 13.823	58.364 ± 12.339
pTNM staging^a^				
I		4	1	0
II		13	9	3
III		6	7	4
IV		5	7	4
Location				
Buccal mucosa	1	4	4	`1
Tongue	4	7	9	5
Gingiva	1	7	3	2
Floor of the mouth	3	6	4	2
The hard palate	2	4	3	0
Retromolar trigone	1	0	1	1

^a^pTNM staging is classified according to the 8th Edition of the UICC TNM Classification of Malignant Tumors

Normal oral mucosal tissues from twelve healthy patients, who needed oral surgery at the Oral and Maxillofacial Surgery Department of the Affiliated (Stomatological) Hospital of Southwest Medical University, were cut at the dimension of 5 × 5 mm from the surgical incision site. The sampling sites of these normal tissues were similar to those of patients with OSCC, including tongue, buccal mucosa, gingiva, floor of the mouth, posterior molars, and the hard palate. These twelve patients were informed in detail about this study, and written informed consent was obtained before surgery. To ensure the accuracy and reliability of our findings, we implemented a stringent assessment for the presence of inflammation in these samples. Histological examinations were performed on all potential normal tissues prior to their inclusion in the study. Tissues showing any histopathological signs of inflammation, including but not limited to increased leukocyte infiltration or tissue edema, were systematically excluded, thereby confounding the comparison between normal and OSCC specimens. The sample size of at least ten subjects in each group, determined by the two-sided test, where the α level was specified to 0.05, demonstrated the power of test more than 80% to detect a 5% difference in the comparisons for the immunoreactive scores (IRS) between groups.

### Immunohistochemistry

Five-μm tissue sections were serially cut and placed onto the silanized slides. The tissue sections were de-paraffinized in xylene twice for 5 min each and rehydrated through graded ethanol. Antigen retrieval was performed by placing the tissue slides in sodium citrate buffer (Maxim Biotechnologies, Fuzhou, China), pH 6.0, for anti-ADAM9 and anti-t-PA antibodies, or in Tris–EDTA, pH 9.0, for anti-CDCP1 antibody with hyperbaric heating for 6 min, as per instructions. After the sections were cooled down at room temperature, they were blocked for the endogenous peroxidase activity by incubation in 3% hydrogen peroxide for 10 min and then placed in 1.5% normal blocking serum (Santa Cruz Biotechnology, Santa Cruz, CA, USA) in phosphate-buffered saline (PBS). Thereafter, a 100-μl aliquot of the primary antibody against ADAM9 (#ab186833; Abcam, Cambridge, UK), CDCP1 (#ab252947; Abcam), or t-PA (#ab157469; Abcam), each of which was diluted at 1:200, 1:200, or 1:1000, respectively, in the antibody dilution buffer (ZSBG-BioTM, Beijing, China), was added onto the section and incubated in a humidified chamber for 60 min at 25 ℃. Subsequently, the section was washed in PBS three times, and a 100-µl aliquot of the secondary antibody (Dako RealTM EnVision, Santa Clara, CA, USA) in the antibody diluent was added and incubated for 30 min at 25 ℃. The color was then developed using 3, 3'-diaminobenzidine (Dako RealTM EnVision) under microscopic control. The section was counterstained in hematoxylin solution for 2 min and immediately placed in 0.1% HCl, followed by saturation with lithium carbonate. After gradient dehydration, the section was air-dried and sealed with 1–2 drops of neutral mounting medium (ZSBG-BioTM). Images were captured by the digital slice scanner (MAGSCANNER KF-PRO-005-EX, Ningbo Kangfeng Biotechnology International Co., LTD., Ningbo, China) under 400 × magnification power at the highest resolution.

### Determination for the immunoreactive score

The IRS were determined by using the ImageJ program (NIH, Bethesda, MD, USA). Prior to grading, each specimen was viewed at 100 × magnification to determine the epithelial and connective tissue orientation, and then graded at 400 × magnification. In normal oral mucosa, scoring was performed only in the epithelial layer, whereas in oral cancer specimens, scoring was performed in cancer cell nests. Each section was first screened, and then three fields of vision were selected to best represent the characteristic of each section.

The IRS were derived from multiplication of the score of the percentage of positively stained cells (0–4)—where 0 = 0%, 1 =  < 10%, 2 = 10–50%, 3 = 51–80%, and 4 =  > 80%—with the intensity score (0–3)—where 0 = negative, 1 = weak, 2 = moderate, and 3 = intense. The scores of each sample were semi-quantitatively assessed by two researchers (L.C. & T.L.). To avoid a recall bias, the same image was re-scored by the same researcher within a one-week interval. The intra-examiner reliability, reported as a kappa value, is 0.970 for ADAM9, 0.978 for CDCP1, and 0.979 for t-PA. If the scores from the two researchers turned out to be significantly different, the decision was made by a third researcher (S.W.).

### Statistical analysis

The differences in ADAM9, CDCP1, and t-PA expressions between OSCC and normal oral tissues were analyzed by the independent sample t-test using GraphPad PRISM version 6.01 (San Diego, CA, USA) with a P value < 0.05 to be regarded as statistically significant.

The correlations between ADAM9, CDCP1, or t-PA expression and tumor pathological differentiation were determined by the Spearman rank correlation, while those between each pair of the three biomolecules were determined by the Pearson correlation. The chi-square test and one-way ANOVA were used to determine the significant difference of clinical features (Table 1), whereas the independent sample t-test and one-way ANOVA were used for univariate analysis (Table 2). Multivariate survival analysis (Table 3) was performed by the proportional risk Cox regression to show the significant 95% confidence interval (CI). The cutoff value (Youden index) of each biomolecule was derived from the time-dependent receiver operating characteristic (ROC) curve. Patients’ overall survival was analyzed using the Kaplan–Meier method and logarithmic rank test. Overall survival is defined as the period of time between the date of surgical operation and the date of patients’ death or the deadline for experimental observation. All statistical tests were performed using SPSS® version 20.0 for IBM^®^ (Chicago, IL, USA), and a P value < 0.05 was considered statistically significant.

**Table 2:** Univariate analysis of ADAM9, CDCP1, and t-PA expressions, demonstrated as the immunoreactive scores (mean ± SD), and clinical variables

Characteristic		n	ADAM9		CDCP1		t-PA	
			Mean ± SD	*P* value	Mean ± SD	*P* value	Mean ± SD	*P* value
Gender	Male	48	8.313 ± 2.769	0.177^a^	7.880 ± 3.443	0.886^a^	9.130 ± 2.856	0.992^a^
	Female	15	7.333 ± 2.257		7.730 ± 2.963		9.130 ± 2.416	
Age	< 60 years	25	7.880 ± 2.666	0.634^a^	8.320 ± 3.449	0.413^a^	8.960 ± 2.441	0.698^a^
	≥ 60 years	38	8.211 ± 2.703		7.630 ± 3.106		9.240 ± 2.945	
pTNM stage	I-II	30	7.063 ± 2.169	0.002^a^*	7.430 ± 3.350	0.356^a^	8.100 ± 2.952	0.004^a^*
	III-IV	33	9.129 ± 2.766		8.210 ± 3.286		10.060 ± 2.179	
Pathological tumor size	≤ 2 cm	10	6.600 ± 2.547	0.020^b^*	6.800 ± 3.615	0.469^b^	6.400 ± 2.875	0.002^b^*
	2 cm < T ≤ 4 cm	39	7.923 ± 2.486		7.870 ± 3.189		9.030 ± 2.433	
	≥ 4 cm	14	9.571 ± 2.709		8.500 ± 3.503		11.360 ± 1.277	
Cervical lymph node metastasis	No	36	7.333 ± 2.541	0.009^a^*	8.030 ± 3.247	0.610^a^	8.750 ± 2.980	0.210^a^
	Yes	27	9.074 ± 2.556		7.590 ± 3.445		9.630 ± 2.339	
Recurrence	No	45	7.778 ± 2.593	0.177^a^	8.240 ± 3.325	0.128^a^	8.980 ± 2.784	0.499^a^
	Yes	18	8.833 ± 2.792		6.830 ± 3.148		9.500 ± 2.662	
Treatment	S	16	7.250 ± 2.910	0.071^b^	7.380 ± 3.649	0.609^b^	7.880 ± 2.964	0.072^b^
	S + C/R	33	7.909 ± 2.213		8.240 ± 3.113		9.330 ± 2.521	
	S + C + R	14	9.429 ± 3.056		7.430 ± 3.502		10.070 ± 2.645	

^a^by independent sample t-test

^b^by one-way ANOVA

*C* Chemotherapy, *R* Radiation therapy, *S* Surgery

^*^denotes the statistically significant difference at P < 0.05

**Table 3:** Multivariate analysis of overall survival in patients with OSCC (n = 63)

Factor	ADAM9 Overall Survival		CDCP1 Overall Survival		t-PA Overall Survival		ADAM9 + CDCP1 Overall Survival		ADAM9 + t-PA Overall Survival		CDCP1 + t-PA Overall Survival		ADAM9 + CDCP1 + t-PA Overall Survival	
	HR (95%CI)	*P* value	HR (95%CI)	*P* value	HR (95%CI)	*P* value	HR (95%CI)	*P* value	HR (95%CI)	*P* value	HR (95%CI)	*P*value	HR (95%CI)	*P* value
Gender														
Male	1		1		1		1		1		1		1	
Female	1.095 (0.258–4.653)	0.902	0.828 (0.213–3.222)	0.785	0.970 (0.265–3.549)	0.964	1.033 (0.240–4.453)	0.965	1.079 (0.252–4.619)	0.918	0.825 (0.209–3.251)	0.783	1.021 (0.235–4.435)	0.977
Age														
<60 years	1		1		1		1		1		1		1	
≥ 60 years	1.123 (0.272–4.639)	0.872	1.487 (0.421–5.250)	0.538	1.581 (0.392–6.380)	0.520	1.122 (0.280–4.495)	0.871	1.085 (0.253–4.653)	0.912	1.414 (0.379–5.278)	0.606	1.096 (0.265–4.535)	0.899
pTNM stage														
I-II	1		1		1		1		1		1		1	
III-IV	2.546 (0.367–17.660)	0.344	1.753 (0.234–13.156)	0.585	2.633 (0.355–19.528)	0.344	2.173 (0.285–16.592)	0.454	2.574 (0.364–18.208)	0.344	1.913 (0.239–15.300)	0.541	2.216 (0.285–17.212)	0.447
Pathological tumor size														
≤ 2 cm	1		1		1		1		1		1		1	
2 cm < T ≤ 4 cm	2.219 (0.466–10.571)	0.317	3.372 (0.714–15.925)	0.125	3.176 (0.645–15.646)	0.155	2.199 (0.450–10.751)	0.330	2.126 (0.444–10.184)	0.345	2.754 (0.526–14.426)	0.231	2.111 (0.425–10.493)	0.361
≥ 4 cm	0.718 (0.091–5.657)	0.753	1.500 (0.216–10.398)	0.681	0.691 (0.074–6.439)	0.745	0.870 (0.101–7.505)	0.899	0.595 (0.060–5.904)	0.658	1.019 (0.106–9.770)	0.987	0.744 (0.067–8.287)	0.810
Cervical lymph node metastasis														
No	1		1		1		1		1		1		1	
Yes	0.283 (0.042–1.894)	0.193	0.530 (0.080–3.519)	0.511	0.358 (0.056–2.293)	0.278	0.365 (0.047–2.839)	0.335	0.283 (0.042–1.894)	0.193	0.475 (0.069–3.291)	0.451	0.357 (0.046–2.789)	0.326
Recurrence														
No	1		1		1		1		1		1		1	
Yes	2.128 (0.201–22.580)	0.531	2.460 (0.234–25.858)	0.453	1.713 (0.172–17.049)	0.646	2.548 (0.228–28.470)	0.447	2.257 (0.207–24.634)	0.504	2.719 (0.254–29.150)	0.409	2.634 (0.232–29.860)	0.434
Treatment														
S	1		1		1		1		1		1		1	
S + C/R	0.264 (0.067–1.035)	0.056	0.251 (0.065–0.979)	0.055	0.253 (0.061–1.052)	0.059	0.273 (0.071–1.060)	0.061	0.269 (0.068–1.058)	0.060	0.265 (0.067–1.042)	0.057	0.277 (0.071–1.079)	0.064
S + C + R	0.317 (0.022–4.461)	0.394	0.404 (0.028–5.913)	0.508	0.713 (0.051–9.906)	0.801	0.260 (0.017–3.922)	0.331	0.320 (0.023–4.499)	0.398	0.385 (0.027–5.583)	0.484	0.267 (0.018–4.015)	0.339
ADAM9														
Low (IRS ≤ 7)	1		–		–		1		1		–		1	
High (IRS > 7)	5.371 (1.158–24.897)	0.032*	–	–	–	–	3.932 (0.652–23.725)	0.135	4.922 (0.992–24.428)	0.051	–	–	3.725 (0.586–23.676)	0.163
CDCP1														
Low (IRS ≤ 7)	–		1		–		1		–		1		1	
High (IRS > 7)	–	–	2.749 (0.858–8.811)	0.089	–	–	1.537 (0.380–6.219)	0.546	–	–	2.534 (0.764–8.403)	0.129	1.501 (0.362–6.230)	0.576
t-PA														
Low(IRS ≤ 10.5)	–		–		1		–		1		1		1	
High(IRS > 10.5)	-	–	–	–	1.955 (0.588–6.501)	0.274	–	–	1.265 (0.364–4.390)	0.712	1.506 (0.460–4.926)	0.498	1.200 (0.346–4.159)	0.774

*CI* Confidence interval, *HR* Hazard ratio, *IRS* Immunoreactive score, *C* Chemotherapy, *R* Radiation therapy, *S* Surgery

^*^denotes the statistically significant difference at *P* < 0.05

## Results

### Significant increases in ADAM9, CDCP1, and t-PA expressions in OSCC

When the gender proportion, mean ages, and biopsy locations were compared between patients with different histopathological diagnoses of OSCC and healthy donors, no significant difference was found between these two study cohorts (Table 1), indicating case–control matching in this study. Moreover, there was no significant difference in pTNM staging among the three different histopathological diagnoses of OSCC (Table and Supplementary Table 1).

**Fig. 1:**
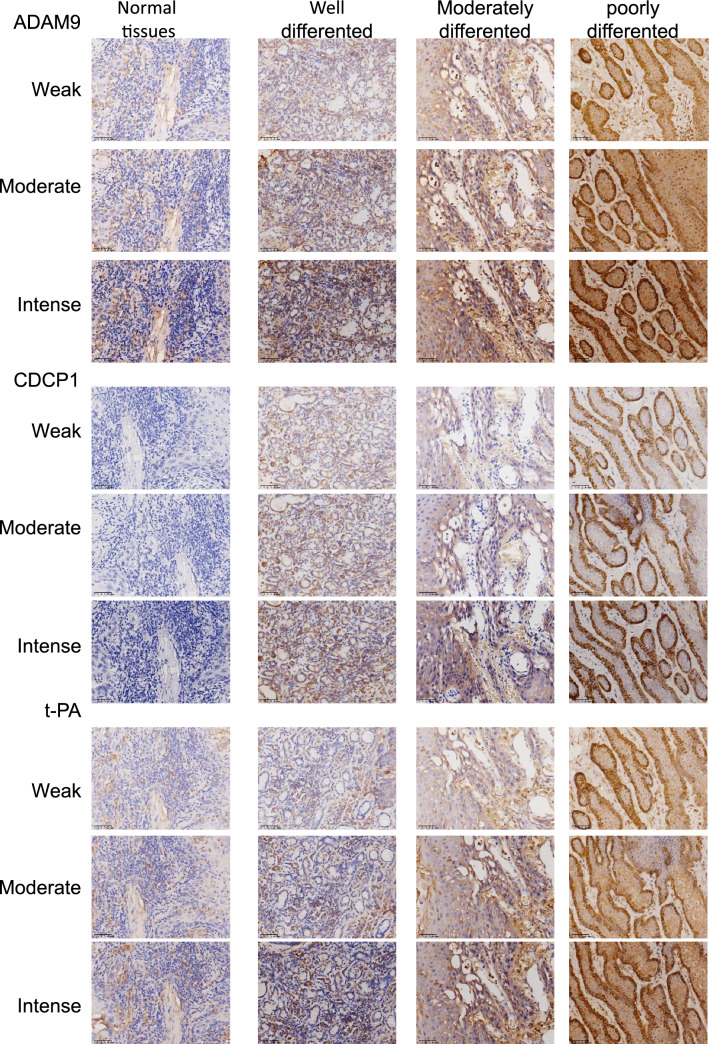
Representative immunohistochemical images of various staining intensities, including weak, moderate, and intense for ADAM9, CDCP1, and t-PA expressions in normal oral tissues and in different histopathological diagnoses, including well, moderately, and poorly differentiated oral squamous cell carcinoma (OSCC) tissues. Bars = 50 μm

**Fig. 2:**
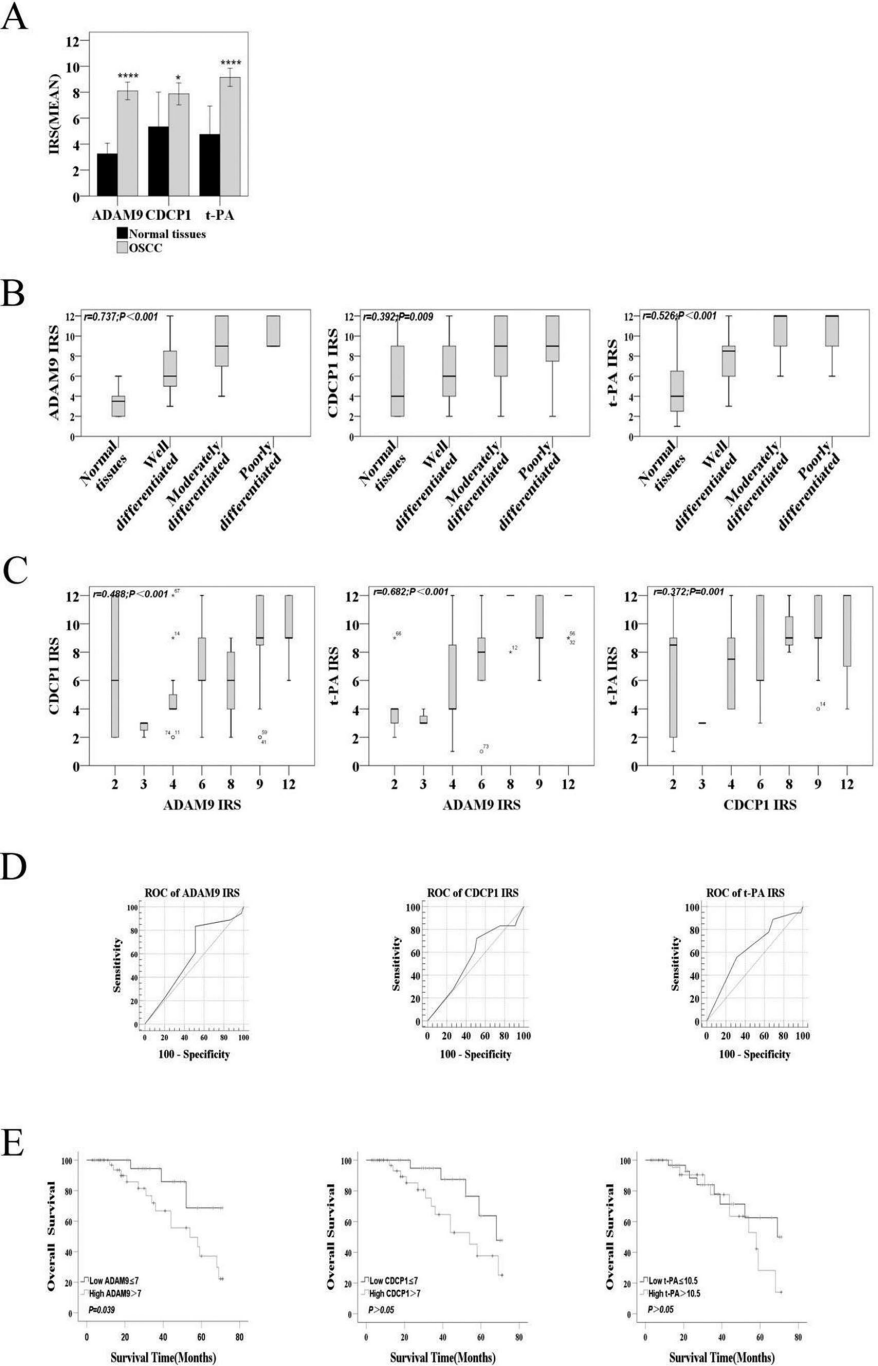
Correlation of ADAM9, CDCP1, and t-PA expression and histopathological differentiation, as well as overall survival in OSCC. (**A**) A bar graph comparing the mean immunoreactive scores (IRS) of ADAM9, CDCP1, and t-PA between 63 OSCC (gray) and 12 normal (black) tissues, expressed as mean ± SD (error bars); Statistical analysis was performed using unpaired student’s t test, *P < 0.05 ****P < 0.0001. (**B**) The correlations between the IRS of ADAM9, CDCP1, or t-PA and the distinct degrees of histopathological differentiation, including 28 well differentiated OSCC, 24 moderately differentiated OSCC, 11 poorly differentiated OSCC, and 12 normal tissues. A horizontal line in each box represents a median with the interquartile range. (**C**) The correlations between each pair of the IRS for ADAM9, CDCP1, and t-PA. A horizontal line in each box represents a median with the interquartile range; an empty circle represents an outlier; an asterisk represents an extreme value. (**D**) The cutoff values for the three biomolecules, including ADAM9, CDCP1, and t-PA, were estimated from the time-dependent receiver operating characteristic (ROC) curves of their IRS. (**E**) The Kaplan–Meier overall survival analysis for 63 patients with OSCC according to the high (gray) and the low (black) IRS of ADAM9, CDCP1, and t-PA during a 4-year follow-up period. Overall survival is defined as a period between the date of surgery and the date of death or the end of research project

Immunohistochemical results showed various staining intensities, ranging from weak to intense, of ADAM9, CDCP1, and t-PA expressions in the cytoplasm or at the cell membrane of most cancer cells within the cancer cell nests of OSCC specimens (Fig. 1). Of the 63 OSCC specimens, 36 (57.1%), 38 (60.3%), and 45 (71.4%) were intensely stained for ADAM9, CDCP1, and t-PA, respectively, whereas only one (8.3%), three (25%), and three (25%) of the twelve normal oral tissues were for ADAM9, CDCP1, and t-PA, respectively. Most of the ADAM9, CDCP1, and t-PA staining patterns were non-specific within the nuclei or weak at the cell membrane of normal oral epithelial cells (Fig. 1). By the independent sample *t*-test, the mean IRS of ADAM9, CDCP1, and t-PA in OSCC were found to be significantly greater than those in normal oral tissues (*P* < 0.01, Fig. 2A).

### ADAM9, CDCP1, and t-PA expressions in relation to histopathological differentiation of OSCC

By the Spearman correlation test, the IRS of ADAM9, CDCP1, and t-PA were significantly and positively correlated with distinct degrees of tumor cell differentiation in OSCC (*r* = 0.737, *P* < 0.001; *r* = 0.392, *P* = 0.009; and *r* = 0.526, *P* < 0.001; respectively; Fig. 2B), suggesting that enhanced expressions of ADAM9, CDCP1, and t-PA are associated with high-grade OSCC. Furthermore, by the Pearson correlation test, there were significantly positive correlations between the IRS of ADAM9 and those of CDCP1 or those of t-PA (*r* = 0.488, *P* < 0.001 or *r* = 0.682, *P* < 0.001; respectively; Fig. 2C). In addition, there was a significantly positive correlation between the IRS of CDCP1 and those of t-PA (*r* = 0.372, *P* = 0.001, Fig. 2C). These findings propose a possible molecular connection among the three biomolecules in OSCC, as previously lung adenocarcinoma [20].

### ADAM9 overexpression in relation to shortened overall survival

By the univariate analysis, the mean IRS of ADAM9 and of t-PA in OSCC were significantly greater in pTNM stages III-IV than those in stages I-II (*P* = 0.002 and *P* = 0.004, respectively; Table 2). The mean IRS of ADAM9 and of t-PA were also significantly greater in OSCC cases with large tumor diameters than in those with small tumor diameters (*P* = 0.020 and *P* = 0.002, respectively). However, only the mean IRS of ADAM9 was significantly higher in OSCC cases with cervical lymph node metastasis than in that without cervical lymph node metastasis (*P* = 0.009). None of the clinical variables listed in Table 2 was found to be significantly associated with the increased mean IRS of CDCP1. Taken together, all of these results imply the association of enhanced ADAM9 expression with progression, growth, and metastasis of OSCC.

By the multivariate analysis, only the ADAM9 overexpression, i.e., IRS > 7, not the CDCP1 or t-PA overexpression, was found to be an independent prognostic marker for 63 OSCC cases with a hazard ratio of 5.371 [95%CI (1.158–24.897), *P* = 0.032, Table 3], implying that the risk of death rises by 5.371 times in the high ADAM9 expression group, compared with the low ADAM9 expression group. However, when the IRS of ADAM9, CDCP1, and t-PA were combined in the multivariate analysis, neither of these combined expressions was found to be an independent prognostic indicator for 63 patients with OSCC (Table 3).

The cutoff value (Youden index) of each biomolecule is derived from the ROC curve (Fig. 2D). The average survival time of the low ADAM9 expression group (IRS ≤ 7) was 62.323 months, whereas that of the high ADAM9 expression group (IRS > 7) was 49.903 months. By the Kaplan–Meier method and log-rank test, an overall survival rate of the low ADAM9 expression group was significantly greater than that of the high ADAM9 expression group (*P* = 0.039, Fig. 2E). The average survival times of the low CDCP1 (IRS ≤ 7) and t-PA (IRS ≤ 10.5) expression groups were 62.056 and 56.909 months, respectively, while those of the high CDCP1 (IRS > 7) and t-PA (IRS > 10.5) expression groups were 49.242 and 51.691 months, respectively. Under the same survival analysis, the low CDCP1 or t-PA expression group was not found to have a significantly greater overall survival rate than the high CDCP1 or t-PA expression group (*P* > 0.05, Fig. 2E).

## Discussion

Several previous studies have shown that ADAM9 is overexpressed in various types of cancer [8, 9, 15, 28–34]. By immunohistochemistry, the expression of ADAM9 protein was also found to be significantly enhanced in OSCC, consistent with the previous results of Tanasubsinn et al. [19]. Moreover, our immunohistochemical findings further revealed significantly increased expressions of CDCP1 and t-PA in OSCC, which correspond with CDCP1 overexpression in renal cell carcinoma[22] and in lung adenocarcinoma [23] and with t-PA overexpression in lung cancer [24]. These three studies [22–24] have additionally demonstrated the clinical significance of CDCP1 and t-PA overexpression on a low patients’ overall survival rate, whereas, in our study, overexpression of CDCP1 or of t-PA in OSCC was not found to be significantly associated with a decreased overall survival rate. In addition, the study of Lin et al. [24] in the lung cancer showed that ADAM9 overexpression mediated t-PA activation to enhance the expression of CDCP1, suggesting that the ADAM9/CDCP1/t-PA pathway plays a synergistic role in the development of lung cancer and that the high expressions of these three biological molecules are associated with poor prognosis of patients. However, the findings from our multivariate analysis in OSCC instead failed to demonstrate the influence of any combined expressions of these three molecules or that of CDCP1 or t-PA expression alone on the prognosis of patients with OSCC, implying a non-existence of this pathway in OSCC. The discrepancy may have been due to distinct molecular pathogenesis between OSCC and other solid tumors, like renal cell carcinoma or lung adenocarcinoma.

Our findings suggest that the expressions of ADAM9, CDCP1, and t-PA play significant roles in the progression of OSCC. Specifically, we observed that elevated levels of these biomolecules were correlated with more aggressive clinicopathological features and poorer patient survival outcomes. These results support our hypothesis that the pathways involving ADAM9, CDCP1, and t-PA are critical in the progression of OSCC. Modulating these pathways could, therefore, offer new therapeutic strategies for managing OSCC. Future studies should focus on exploring targeted interventions that could inhibit the overexpression of ADAM9 and t-PA, and further investigate the underexplored role of CDCP1 in OSCC progression. By understanding and potentially disrupting these pathways, we may be able to develop more effective treatments that improve patient prognosis and reduce the overall burden of this disease.

The results by Spearman correlation test showed that overexpression of ADAM9, CDCP1, or t-PA in OSCC was positively correlated with distinct degrees of tumor cell differentiation, as defined by different histopathological diagnoses. Generally, the poorly differentiated OSCC, in comparison with the well differentiated or the moderately differentiated OSCC, is associated with cancer aggressiveness due to its high recurrence rate and low disease-free survival [35]. Accordingly, the median IRS of ADAM9, CDCP1, and t-PA were found to be greater in the poorly differentiated OSCC than in the well differentiated OSCC or in normal oral tissues. The strongly positive correlation between the median ADAM9 IRS and distinct grades of OSCC differentiation may be involved in mediating the transition from the well differentiated OSCC to the moderately differentiated OSCC, and then to the poorly differentiated OSCC, probably resulting in enhanced aggressiveness of oral cancer cells, as proposed by Peduto et al. [36] for a crucial role of ADAM9 expression in the transition from the well differentiated to the poorly differentiated prostate cancer. The median IRS of CDCP1 and t-PA were also found to be positively, albeit moderately, correlated with the distinct degrees of OSCC differentiation, suggesting that CDCP1 and t-PA may be associated with aggressiveness of oral cancer as well.

Nevertheless, the findings from our univariate analysis revealed that only was the mean IRS of ADAM9 significantly increased in OSCC specimens with pTNM stages III and IV, large tumor size, and the presence of cervical lymph node metastasis, which is in line with our previous in vitro finding that demonstrated involvement of ADAM9 with oral cancer cell invasion [37]. Moreover, t-PA expression in OSCC was significantly enhanced in advanced pTNM stages and large tumor size, but not with the presence of cervical lymph node metastasis, whereas CDCP1 expression in OSCC was unaffected by any clinical variables. All of these findings from the Spearman correlation test, the univariate analysis, and the aforementioned multivariate analysis suggest that, unlike the lung adenocarcinoma, the ADAM9/CDCP1/t-PA pathway may not exist in OSCC, despite the significantly positive correlations found among the three molecules by the Pearson correlation test. A rising trend for the mean IRS of ADAM9 or of t-PA in the OSCC specimens of patients, who required two or three therapeutic combinations, is noteworthy although these increased means did not reach the significance level.

The Kaplan–Meier survival analysis showed that although the high ADAM9, CDCP1, or t-PA expression group led to a shorter survival time and worse prognosis than the low expression group, only the ADAM9 overexpression was significantly correlated with the shortened survival time, the low overall survival rate, and the poor prognosis of patients with OSCC. As a result, it is suggested by this study that the degree of ADAM9 expression alone could be used as an independent target for the prognosis of patients with OSCC, whereas the expression of CDCP1 or that of t-PA is neither significant for patients’ prognosis nor regarded as an independent prognostic marker of OSCC. Consistently, among several salivary proteases, elevated ADAM9 levels are indeed considered a biomarker for OSCC screening and diagnosis [38], whereas an up-regulation of t-PA expression is not obviously found in oral cancer cells in comparison with adjacent non-malignant epithelial cells [39]. The findings from the survival analysis in this study are not consistent with those in renal cell carcinoma [22] and lung adenocarcinoma [23] and may again verify the non-existence of the ADAM9/CDCP1/t-PA pathway in OSCC.

In conclusion, this study demonstrated overexpression of ADAM9, CDCP1, and t-PA in OSCC, which were positively correlated with tumor cell differentiation of OSCC. Furthermore, this study revealed that only the high degree of ADAM9, but not CDCP1 or t-PA, expression in OSCC was significantly related to a shortened survival time, reduced overall survival, and then poor prognosis of patients with OSCC. Inhibition of ADAM9 expression in OSCC may thus have a therapeutic effect on improvement of the patients’ prognosis by extending their survival time and improving their overall survival rate. Note that the neutralizing monoclonal antibody raised against the disintegrin and the cysteine-rich domains of ADAM9, involved in human keratinocyte migration [40], is now being developed and tested. Moreover, ADAM9 expression is an independent prognostic marker of OSCC, which can provide a reference and a strong basis for ADAM9 as a molecular target for targeted therapy of OSCC. Finally, as the specific regulatory and influencing factors of the ADAM9/CDCP1/t-PA pathway in OSCC are still poorly understood, it is interesting to further explore them in subsequent studies.

In our research, while the primary focus was on the parameters defined by the pTNM staging system, we acknowledge the significance of other histopathological risk factors such as invasion patterns, perineural invasion, and extracapsular spread. These factors, although not directly integrated into our core analytical approach, hold the potential to deepen our understanding of tumor behavior. Incorporating these additional histopathological risk factors alongside the established pTNM criteria in future studies could pave the way for a more nuanced risk assessment. This integration has the potential to enhance the prognostication of diseases and refine treatment decision-making processes, offering a broader perspective on patient management.

## Supplementary Information

Additional file 1

## Data Availability

The data that support the findings of this study are available on request from the corresponding author.

## Abbreviations

ADAM9: A disintegrin and metalloproteinase 9
OSCC: Oral squamous cell carcinoma
CDCP1: CUB domain-containing protein 1
t-PA: Tissue-type plasminogen activator
IRS: Immunoreactive scores
